# Periodontium-derived fibroblasts as a model to evaluate inflammation and pharmacological modulation of osteogenesis and osteoclast formation

**DOI:** 10.3389/fcell.2026.1813291

**Published:** 2026-05-01

**Authors:** Teun J. de Vries, Merve Ceylan, Ton Schoenmaker, Marja L. Laine

**Affiliations:** 1 Department of Oral Cell Biology, Academic Centre for Dentistry Amsterdam, University of Amsterdam and Vrije Universiteit, Amsterdam, Netherlands; 2 Department of Periodontology, Academic Centre for Dentistry Amsterdam, University of Amsterdam and Vrije Universiteit, Amsterdam, Netherlands

**Keywords:** 3D cell models, cell models for periodontal diseases, gingiva fibroblasts, osteoclast, osteogenesis, periodontal ligament fibroblasts (PDLFs)

## Abstract

Two anatomically and functionally distinct populations of periodontium-derived fibroblasts can be identified: gingiva fibroblasts (GFs), which originate from the soft connective tissue of the gingiva and support epithelial attachment and tissue integrity, and periodontal ligament fibroblasts (PDLFs), which produce collagenous fibers that anchor teeth within the alveolar bone socket. Both cell types can contribute to osteogenesis when cultured in the presence of mineralization medium, and both cell types can drive osteoclast formation when co-cultured with osteoclast precursors. Under inflammatory conditions such as periodontitis, this balance is disturbed, leading to more osteoclast-driven bone resorption. The model system of osteogenesis and osteoclastogenesis can be used to further dissect the contributing factors of periodontitis by incorporating bacteria or their components like TLR agonists. Chronic inflammation can further be mimicked by using inflammatory cytokines such as IL-1β, TNF-α and Activin-A. Furthermore, bone anabolic and (anti-)catabolic medications such as parathyroid hormone (PTH), anti-TGF-β, sclerostin and anti-sclerostin can be used to investigate their effects on both osteogenesis and osteoclastogenesis. This platform is ideal for studying the effect of medication used in comorbidities of periodontitis, such as rheumatoid arthritis (RA; e.g., anakinra, infliximab) and diabetes (e.g., metformin), which have all been shown to inhibit osteoclast formation. The osteogenesis culture system can be manipulated over time, making it an ideal system for studying how the osteogenic stage of periodontium-derived fibroblasts affects subsequent osteoclast formation. Finally, fibroblast-based three-dimensional (3D) culture systems provide physiologically relevant environments that capture spatial cell-matrix interactions essential for hard and soft tissue repair in periodontal tissue regeneration. Collectively, these models help bridge *in vitro* findings toward optimized regenerative strategies.

## 1 Introduction

The body’s interfaces with the external environment, such as the skin, the oral cavity, and the intestine, are lined by epithelium. This tissue type forms an effective barrier against microbial invasion through tight cell–cell junctions. Teeth represent a notable exception to this general barrier principle. In the oral cavity, the gingival epithelium attaches to the tooth surface and forms a tight seal, which can become vulnerable to inflammation, particularly under conditions of inadequate oral hygiene. Beneath the oral epithelium, two anatomically and functionally connective tissues can be distinguished. First, the gingiva, in which fibroblast-produced collagen fibrils connect the epithelium to the alveolar bone and tooth. The second, anatomically distinct connective tissue is the periodontal ligament, that forms the approximately 0.1 mm thin ligament that anchors teeth into the alveolar bone of the tooth socket (Beertsen et al., 1997).

**FIGURE 1:**
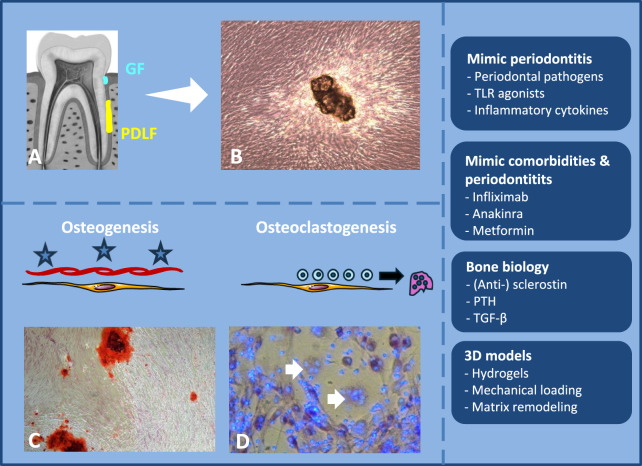
Illustration and synopsis of the manuscript. **(A)** Anatomical location of the periodontium-derived GFs (light blue) and PDLFs (yellow). **(B)** Outgrowth of cells from a tissue fragment. **(C)** Microscopic view of alizarin red staining, staining calcium deposition. **(D)** Micrograph of multinucleated osteoclasts (yellow arrows) in a co-culture of PDLFs and PBMCs. Right column shows the four topics that are discussed. Footnote: GF = gingival fibroblast, PDLF = periodontal ligament fibroblast, PBMC = peripheral blood mononuclear cell. Figure A is from freepic.com.

Both gingiva fibroblasts (GFs) and periodontal ligament fibroblasts (PDLFs) can easily be obtained from extracted teeth. GFs are typically isolated from the coronal attached gingival tissue, whereas PDLFs are retrieved from the middle one-third region of the tooth root (Figure 1A) (Arceo et al., 1991; de Vries et al., 2006). Cells can subsequently be expanded through outgrowth from tissue fragments (Figure 1B), and cultures at passage five are commonly used for biological assays. Since the 1980s, both fibroblast types have been characterized to some extent (Somerman et al., 1988; Mariotti and Cochran, 1990; de Vries et al., 2006). Initial studies suggested functional differences between these populations. GFs were reported to proliferate faster and to produce relatively more non-collagenous proteins (Mariotti and Cochran, 1990), while PDLFs had more alkaline phosphatase (ALP) activity (Somerman et al., 1988; Arceo et al., 1991). In addition, PDLFs were shown to deposit mineral (Figure 1C). Notably, the extent of mineral deposition did not correlate with the level of ALP activity (Arceo et al., 1991). Already in these initial studies, it was shown that there was a huge variation between different donors (Somerman et al., 1988; Arceo et al., 1991), which is repetitively confirmed in later studies.

Next to the discovery in the 1980s that PDLFs, and to a lesser extent GFs, can contribute to osteogenesis, Kanzaki et al. showed that PDLFs can orchestrate osteoclast formation when seeded with peripheral blood mononuclear cells (PBMCs) in direct cell-cell contact cultures (Figure 1D). In this setting, PDLFs were shown to express receptor activator of NF-kB ligand (RANKL), an important differentiation factor for osteoclast formation, as well as the inhibitor of this factor, osteoprotegerin (OPG) (Kanzaki et al., 2001). In subsequent work, PDLFs were found to induce osteoclast formation more effectively than GFs (de Vries et al., 2006). Notably, no osteoclasts formed in the absence of fibroblasts (de Vries et al., 2006; Moonen et al., 2018). PDLFs were also superior in inducing osteoclast formation in assays comparing them to alveolar bone cells from the same patient (Loo-Kirana et al., 2021). Next to cell-cell inductions of periodontium-derived fibroblasts, osteoclasts are also induced after paracrine secretion of osteoclastogenesis contributing cytokines by GFs (Novello et al., 2024).

A recent study has challenged the concept that PDLFs differ extensively from GFs (Shetty et al., 2025), showing that GFs also have a similar capacity to differentiate into the osteogenic lineage. Although this review uses the term fibroblast, it is important to note that both outgrowths of periodontium-derived tissues are rich in mesenchymal stem cells. This was shown for gingival tissue-derived cells by the high expression of CD-markers associated with mesenchymal stem cells such as CD73, CD44, CD29 and CD90, which were present in nearly 100% of the cells. It was further shown that these cells demonstrated tri-lineage commitment into chondrogenesis, adipogenesis and osteogenesis, which is a prime characteristic for mesenchymal stem cells (Zhang et al., 2019). Similarly, the presence of stem cells within the periodontal ligament has recently been demonstrated in the perivascular areas of the murine PDL (Men et al., 2020). Like with human GFs, human PDL outgrowths contained high percentages of cells that are positive for mesenchymal stem cell markers CD73, CD90 and CD105 (Behfarnia et al., 2023).

Although the term “fibroblast” is used throughout this review, it is important to recognize that periodontium-derived fibroblasts represent a heterogeneous cell population with mesenchymal stem cell (MSC)-like characteristics. Gingival and periodontal ligament fibroblasts have been shown to express canonical MSC markers and exhibit multilineage differentiation capacity, including osteogenic, chondrogenic, and adipogenic potential (Parisi et al., 2024). However, rather than representing a uniform MSC population, these cells retain tissue-specific functional identities that are closely linked to their anatomical origin.

A new level of understanding fibroblast diversity in the oral tissues comes from single-cell RNA sequencing (scRNA-seq) studies, which provide direct insight into cell populations present *in situ*. Williams and coworkers (Williams et al., 2021) identified three different fibroblast types in the soft periodontal tissue. Archetypical fibroblasts, whose main function is to make collagen type I and matrix metalloprotease 2 could be distinguished as well as those that have a signature of leukocyte proliferation, granulocyte migration (CXCL1,2,8) and complement activation (C3) (Williams et al., 2021). Similarly, a recent study compared the cell composition of human gingiva and periodontal ligament tissues (Zhu et al., 2026). With the exception of epithelial cells, the overall composition of cell types present in these tissues was comparable. Interestingly, this study also distinguished different types of fibroblasts *in situ*, with various functions such as collagen synthesis and cell-cell interactions (Zhu et al., 2026). When comparing scRNA-seq gingival fibroblast populations from periodontitis tissue with cultured fibroblasts, it was confirmed that various fibroblasts can be identified with scRNA-seq *in situ,* but in addition, various fibroblast populations are still present after culture (Wu et al., 2025). Interestingly, although the mesenchymal stem cell characteristics of cells within periodontal tissues are well acknowledged, such populations are not predominantly identified as distinct clusters in scRNA-seq analysis and may instead be embedded within the broader fibroblast populations (Williams et al., 2021; Wu et al., 2025; Zhu et al., 2026). Together, these findings support the concept that periodontium-associated fibroblasts represent a heterogeneous population with stem/progenitor-like features rather than a discrete MSC population, retaining tissue-specific functional identities despite sharing MSC-associated markers and differentiation capacity.

## 2 Mimicking periodontitis with periodontal pathogens, their components and inflammatory cytokines

Although many studies focus on inflammatory responses of leukocytes in diseases such as periodontitis, resident fibroblasts can also be evoked to elicit an inflammatory response. Both GFs and PDLFs respond to periodontal bacteria by producing pro-inflammatory mediators. For example, exposure to *Actinobacillus actinomycetemcomitans* induced expression of interleukin (IL)-1β, IL-6, and tumor necrosis factor-α (TNF-α) in GFs (Belibasakis et al., 2005). Similarly, *Porphyromonas gingivalis* triggered inflammatory responses in both PDLFs and GFs (Scheres et al., 2010). Scheres et al. compared the responses of GFs and PDLFs and demonstrated substantial inter-donor variability but overall broadly comparable responses from both fibroblasts in terms of expression of IL-1β, IL-6, IL-8, and TNF-α. In addition, macrophage colony stimulating factor (M-CSF) expression was induced by *P*. *gingivalis* (Scheres et al., 2010). Since the phenotype of GFs and PDLFs could be altered in periodontitis, it was addressed whether gene expression of these fibroblasts was different when isolated from patients. It was shown that PDLFs derived from periodontitis patients had higher expression of Toll-like receptors (TLR1, TLF4, and TLR7, pathogen recognition receptors) compared to non-periodontitis control fibroblasts. GFs, on the other hand, responded stronger after an exposure to *P*. *gingivalis* with increased expression of TLR1, TLR2 and TLR7. Interestingly, there was no difference in induction of gene expression after *P. gingivalis* exposure between controls and periodontitis-derived fibroblasts (Scheres et al., 2011).

One could hypothesize that these intrinsic differences between fibroblast populations, and their inflammatory activation by periodontal pathogens, may influence pronounced osteoclast formation. However, priming of PDLFs with *P. gingivalis* for 6 h led to 69% *lower* numbers of osteoclasts than non-exposed controls, whereas the patient-derived PDLFs did not respond with lower osteoclast numbers (Sokos et al., 2014). This suggests that PDLFs from periodontitis patients may have been primed or sensitized *in vivo* prior to isolation. It should be noted that the above-mentioned studies were all with short exposure of up to 6 h to *P. gingivalis* followed by washing away the bacterium thoroughly.

Instead of short priming with bacteria, periodontium-derived fibroblasts could be primed with inflammatory cytokines for a short period to model acute inflammatory activation, as investigated by Bloemen et al. (2011). In brief, PDLFs were exposed to IL-1β for 6 h, followed by washing and removal of the cytokine. Subsequent RNA analysis showed that even this short exposure resulted in prolonged biological effects. Immediately after exposure, adhesion molecule ICAM-1 was highly upregulated as well as IL-1β and M-CSF. This created a beneficial milieu for osteoclast formation: adhesion of PBMCs to PDLFs was increased as well as the ultimate osteoclast formation (Bloemen et al., 2011). Instead of priming with an inflammatory cytokine, PDLFs could also be primed with sera from periodontitis patients, thus containing a clinically relevant mix of inflammatory cytokines. Recently, we performed a study where PDLFs were primed for 3 days with sera from periodontitis patients before and 1 year after successful treatment. This priming did not affect osteoclast formation, likely because the sera per individual before and after treatment were stunningly comparable for monocyte chemoattracting protein-1 (MCP-1), IL-6 and TNF-α (de Vries et al., 2026).

In order to mimic the chronic inflammatory state characteristic of periodontitis, it is difficult or impossible to maintain cultures with viable bacteria. A way to mimic chronic exposure, is to make use of bacterial compounds, such as TLR2 and TLR4 agonists that can be added to osteogenesis and osteoclastogenesis cultures (Karlis et al., 2020). Chronic exposure to TLR agonists in GFs-PBMCs co-cultures led to a decrease in osteoclast formation, whereas osteoclast formation and resorptive activity using monocultures of monocytes induced with M-CSF and RANKL was not affected. Regarding osteogenesis, GFs responded to TLR stimulation with decreased cell proliferation, and TLR4 agonist caused a reduced ALP activity (Karlis et al., 2020).

Another way to mimic the chronic state of inflammation is to culture with cytokines associated with inflammation such as Activin-A (Schoenmaker et al., 2019) or IL-1β (Steemers et al., 2025). Co-cultures of PDLFs from controls or from patients with fibrodysplasia ossificans progessiva (FOP), resulted in fewer osteoclasts, concomitant with lower expression of M-CSF and DC-STAMP (Schoenmaker et al., 2019). Likewise, osteoclasts that were cultured from monocytes in the presence of M-CSF and RANKL and Activin-A were also sparser, but when cultured with Activin-A, the osteoclasts were larger and more active (Schoenmaker et al., 2020), and differentially expressed genes associated with larger osteoclasts (Schoenmaker et al., 2023).

One could also mimic chronic inflammation by adding inflammatory cytokines continuously. Continuous co-culturing of PDLFs and PBMCs in the presence of IL-1β resulted in more and larger osteoclasts. Preceding this higher number of osteoclasts, was the marked presence of leukocyte clusters, only in the cocultures that contained IL-1β (Steemers et al., 2025). Similar to the effects observed after short-term incubation of IL-1β (Bloemen et al., 2011), the chronic exposure resulted in an increase of IL-1β expression as well as an increase in RANKL expression (Steemers et al., 2025). No effects of IL-1β were observed in ALP activity in PDLFs osteogenesis experiments (Steemers et al., 2025).

## 3 Periodontium-derived fibroblasts to study comorbidities of periodontitis

Periodontitis is increasingly recognized as part of a broader systemic disease network and is strongly associated with comorbidities such as RA and diabetes (Hajishengallis, 2022). Importantly, medication that inhibits disease progression of these diseases, could be beneficial for periodontitis as well. Anti-TNF-α treatment is one of the successful treatments for RA (Caporali et al., 2009). Although anti-TNF-α agents cannot be prescribed by dentists, anti-TNF-α agents have been reported to exert beneficial effects on periodontal status (Zamri and de Vries, 2020). Anti-TNF-α medication, infliximab, was used in a study that investigated osteoclast formation using PDLFs from controls and from periodontitis patients. Infliximab did not influence osteoclast formation in these co-cultures, despite induced secretion of TNF-α in such co-cultures. In contrast, in the absence of fibroblasts, infliximab prevented the formation of PBMC clusters and subsequent osteoclast formation (de Vries et al., 2016). Another clinically used medication for RA patients is anakinra, an IL-1β receptor antagonist (Mertens and Singh, 2009). *In vitro*, anakinra inhibited the formation of PBMC clusters that formed in the presence of IL-1β and effectively blocked the role of IL-β in the formation of osteoclasts (Steemers et al., 2025).

Diabetes is another major comorbidity of periodontitis. Metformin is the most frequently used medication to reduce blood glucose levels. It has also been shown to lower osteoclast formation in a co-culture using PDLFs, together with lower gene expression of M-CSF and RANKL. In addition, metformin inhibited osteoclast formation and activity in PBMC cultures both in the presence and absence of exogenous M-CSF and RANKL. Notably, osteogenesis was not affected by metformin (Tao et al., 2021).

## 4 Periodontium-derived fibroblasts and their contribution to understanding bone biology

It is well established that cells from the osteoblast lineage provide essential signals such as RANKL to osteoclast precursors, thereby providing the signal to differentiate into osteoclasts (Udagawa et al., 2021). The degree of signaling is likely dependent on the osteogenic differentiation stage of the cells. We recently addressed this concept by priming PDLFs (Prins et al., 2024) or GFs (Ceylan et al., 2024) with osteogenic medium for 0, 1, 2, or 3 weeks, followed by an osteoclastogenesis assay for an additional 3 week period. In both studies, osteoclastogenic potential progressively declined with the duration of the osteogenic preconditioning. This was accompanied by reduced expression of the osteoclast fusion marker DC-STAMP. Together, these studies indicate that the non-osteogenically stimulated cells provide stronger stimulus for osteoclast formation than their osteogenically differentiated counterparts.

An intriguing feature of the periodontium-derived fibroblast model is that osteoclast formation occurs despite at least 100x higher levels of OPG compared to RANKL (Sokos et al., 2015). In a study using PDLFs derived from FOP patients and controls, we compared the effect of a transforming growth factor-β (TGF-β) inhibitor on osteogenesis and osteoclastogenesis. The rationale behind it was that TGF-β inhibition could result in less osteogenesis, since TGF-β signaling is involved in the pathogenesis of FOP (MacFarlane et al., 2017). TGF-β is one of the key proteins that can steer osteoclast formation in the absence of RANKL (Kim et al., 2005). When inhibiting TGF-β signaling, the PDLFs-mediated osteoclast formation was inhibited in co-cultures of both control and FOP (de Vries et al., 2018), suggesting that TGF-β rather than RANKL could be the driver of osteoclast formation in periodontium-derived fibroblast-mediated osteoclast differentiation.

The PDLFs differ from osteocytes and osteoblasts in terms of their susceptibility to sclerostin, the inhibitor of bone formation. Neither sclerostin nor its bone formation promoting antagonist, rhomosozumab, affected PDLFs-mediated osteogenesis or osteoclastogenesis. This was likely due to a 45.000-fold lower expression of sclerostin in PDLFs compared to *in situ* osteocytes and at least 100-fold lower expression of the receptors involved in sclerostin signaling (Pigeaud et al., 2023).

Another clinically relevant bone anabolic marker is parathyroid hormone (PTH), which is widely used in osteoporosis treatment. It is known to exert both anabolic and catabolic effects. Using PDLFs, anabolic effects were observed in some of the PDLF donors. Co-cultures of PBMCs and PDLFs in the presence of PTH resulted in the formation of larger osteoclast (Talbi et al., 2025).

## 5 3D models of periodontium-derived fibroblasts and periodontal tissue regeneration

The ultimate goal of periodontal regeneration is to restore both the structure and function of periodontal tissues lost due to periodontitis, including the formation of new cementum, alveolar bone, and a functionally oriented PDL (Tassi et al., 2017). This process is highly complex, involving multiple resident and recruited cell types, growth and differentiation factors, signaling networks, and dynamic cell-cell and cell-matrix interactions. Consequently, periodontal regeneration must be investigated in a spatially and temporally coordinated manner (Weinreb and Nemcovsky, 2015). In this context, three-dimensional (3D) *in vitro* culture systems represent a valuable alternative to traditional two-dimensional (2D) models and can complement and ultimately replace animal studies by more closely recapitulating the structural, biochemical, and mechanical features of native extracellular matrix (ECM). By providing a physiologically relevant microenvironment, 3D systems help bridge the gap between mechanistic *in vitro* research and clinical translation (Duval et al., 2017; AlFatlawi et al., 2023).

**FIGURE 2:**
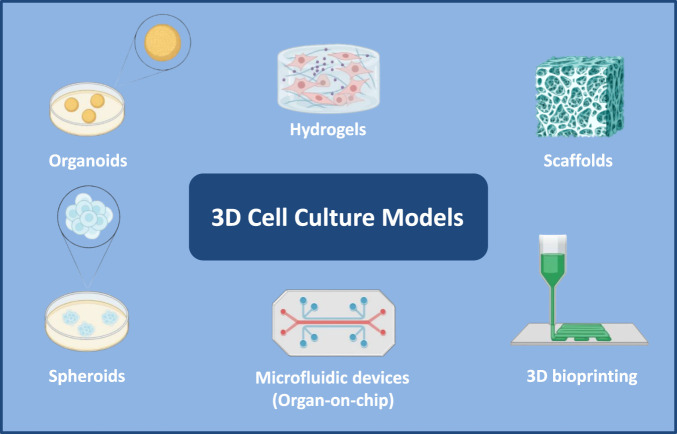
Overview of three-dimensional (3D) cell culture models applied in periodontal research. Schematic representation of major 3D culture platforms used to model oral and periodontal tissues, including organoids, spheroids, hydrogel-based systems, scaffold-based constructs, microfluidic devices (“organ-on-a-chip”), and 3D bioprinting approaches. These platforms provide physiologically relevant microenvironments that better recapitulate extracellular matrix (ECM) architecture, spatial cell–cell and cell–matrix interactions, and mechanical cues compared with conventional two-dimensional cultures. By enabling controlled investigation of fibroblast behavior, osteogenesis, osteoclastogenesis, and tissue remodeling, 3D systems serve as powerful tools to study periodontal hard and soft tissue regeneration and to bridge mechanistic *in vitro* research toward translational applications. Created by https://www.biorender.com/.

Current 3D culture platforms, including organoids, spheroids, hydrogel- and scaffold-based constructs, and microfluidic devices (Figure 2), significantly advanced oral biology research by enabling the study of tissues in more complex, physiologically relevant settings (Perales et al., 2025). Spheroids were among the earliest 3D systems introduced to investigate cell-cell interactions and early tissue formation, representing a key advance in oral biology (Eltom et al., 2019). These 3D cellular aggregates, composed of outer and inner cell layers, create a microenvironment that more closely resembles *in vivo* conditions than traditional monolayer cultures. With the evolution of tissue engineering, more advanced 3D systems were developed to model dental tissues such as gingiva and periodontal ligament (Perales et al., 2025). Scaffold-based platforms using biomaterials like hydrogels and synthetic polymers enabled controlled growth and differentiation of dental cells, supporting dental pulp regeneration and bioengineered tooth constructs (Lee and Mooney, 2001). The development of stem cell–derived dental organoids has further enhanced understanding of tooth development and disease. Integration of microfluidics led to physiologically relevant “tooth-on-a-chip” systems, while combining patient-derived cells with 3D printing enabled personalized dental models (Danku et al., 2022). More recently, bioprinting technologies have facilitated fabrication of complex dental tissues with defined architecture and function, expanding the translational potential of regenerative dental research. Collectively, these systems facilitate improved assessment of cell-ECM interactions and provide models to investigate key regenerative events underlying periodontal hard and soft tissue healing. Because tissue engineering success depends strongly on cellular responses to local microenvironmental cues, understanding how periodontal cells behave within relevant 3D matrices is critical for optimizing regenerative strategies (Perales et al., 2025). Examples of both periodontal soft and hard tissue remodeling using these platforms are discussed below.

A representative example of biomimetic modeling was provided by Oortgiesen et al., who developed a 3D PDL culture system designed to reproduce the anatomical PDL space and allow controlled mechanical loading (Oortgiesen et al., 2012). PDLFs were embedded in a 3D collagen matrix within an engineered “artificial PDL” compartment and exposed to mechanical stimulation, biochemical stimulation using enamel matrix derivative (Emdogain; EMD), or both. Under static conditions, PDLFs remained randomly distributed; however, cyclic stretching increased cell numbers and promoted directional cellular alignment, with fibroblasts orienting predominantly perpendicular to the loading axis, resembling the organized architecture of native PDL tissue. EMD further enhanced proliferative and matrix-associated responses and modulated gene expression, including increased bone sialoprotein (BSP) and collagen type I (COL1) with decreased Runt-related transcription factor-2 (RUNX2), suggesting combined osteogenic and remodeling-associated effects rather than a pure ligament-like phenotype. Notably, while the platform allowed combined stimulation, dual treatment of EMD and mechanical stimulation did not yield a clear synergistic response compared with single-stimulus conditions, highlighting the complexity of pathway interactions governing PDLF behavior. Overall, this model represents a reproducible and physiologically relevant system to study mechanotransduction and regeneration-associated remodeling under defined dynamic loading.

Beyond their central role in ligament regeneration, PDLFs also contribute to periodontal hard tissue healing and display osteogenic plasticity *in vitro*. Alves et al. investigated whether a 3D collagen type I microenvironment modulates osteogenic differentiation of human PDLFs compared to 2D culture (Alves et al., 2015). Following osteogenic induction, 3D collagen altered differentiation outcomes in a time-dependent manner and promoted increased proliferation and enhanced expression of multiple osteogenic and matrix-related markers, including RUNX2, ALP, COL1, and osteopontin (OPN). Importantly, mineralized matrix formation was detectable in 3D cultures, supporting the concept that collagen-based 3D systems promote differentiation-associated features relevant to mineralized tissue regeneration. These findings align with the physiological PDL niche, where fibroblast subpopulations at cementum and bone interfaces exhibit greater mineralization potential, while cells in the central ligament region maintain a predominantly fibroblastic phenotype.

Similarly, Inanc et al. compared osteogenic differentiation of PDLFs cultured in 2D monolayers versus 3D mineralized porous poly (DL-lactic-co-glycolic acid) (PLGA) scaffolds under static or dynamic conditions using a slow-turning lateral bioreactor (Inanc et al., 2006). Osteogenic differentiation occurred in both 2D and 3D conditions; however, scaffold-based cultures expressed higher levels of osteogenic marker proteins including osteonectin (ON), OPN, BSP, and osteocalcin (OCN), particularly in regions of high cell density and ECM deposition. Dynamic culture further increased OCN protein expression compared with static 3D conditions, supporting the concept that shear-related cues can enhance osteogenic maturation. Together, these studies emphasize that 3D scaffold environments can modulate PDLF phenotype and improve spatially regulated mineralization patterns. One of the challenges for 3D models using PDLFs is to mimic spatially relevant osteogenesis. For building up alveolar bone, osteogenesis is only functional when PDLFs deposits bone onto the remaining alveolar bone, whereas the periodontal space ligament should be kept unmineralized to maintain its function.

In parallel with hard tissue regeneration, periodontal regeneration also requires stable soft tissue healing. Gingival and mucosal tissues serve as the primary barrier against the oral microorganisms and are essential for protecting underlying tissues during healing. Makkar et al. investigated how ECM stiffness regulates the inflammatory phenotype of GFs and immune homeostasis (Makkar et al., 2026). Using a collagen-alginate interpenetrating network hydrogel with independently tunable stiffness but constant ligand density, the authors generated “soft” (∼0.75 kPa) and “stiff” (∼2 kPa) matrices reflecting the rheological properties of diseased and healthy gingiva, respectively. In stiff matrices, GFs upregulated ECM synthesis and organization, including collagen fibril formation, with increased expression of COL5A3, COL4A1/2, COL6A5, and TIMP3, consistent with a matrix-preserving phenotype. In contrast, soft matrices induced inflammatory chemokines (CXCL12, CXCL5/6, CCL8, CSF3) and matrix-degrading enzymes (MMP3, MMP12, ADAMTS14). Upon TLR2 stimulation, fibroblasts in soft hydrogels secreted markedly higher levels of IL-6, IL-8, and CCL2, whereas increasing stiffness progressively suppressed inflammatory output. RNA sequencing indicated that this stiffness-dependent suppression was primarily mediated via downregulation of the non-canonical NF-κB pathway. Mechanistically, matrix stiffness altered the ECM-cytoskeletal-nuclear axis: GFs in soft matrices exhibited elongated morphology, prominent actin stress fibers, and enlarged nuclei, whereas stiff matrices induced a more compact phenotype with increased nuclear sphericity. To assess stromal-immune crosstalk, a 3D co-culture system combining GFs with CD34^+^ hematopoietic stem cell-derived myeloid progenitors was developed. Stiff matrices promoted differentiation toward HLA-DR^+^CD11b^+^ dendritic cells with enhanced PD-L1 expression and phagocytic capacity, suggesting an immunomodulatory phenotype. In contrast, soft matrices generated a pronounced pro-inflammatory cytokine milieu. Collectively, these findings have shifted the paradigm of periodontitis from a purely microbe-driven condition toward a model in which ECM softening itself amplifies inflammation, highlighting the therapeutic potential of biomaterials aimed at restoring gingival mechanical integrity.

In another study, Chaussain Miller et al. (2002) compared GFs and dermal fibroblasts using free-floating 3D collagen gels to assess wound-healing–related matrix remodeling. Although overall contraction and cell numbers were comparable, GFs exhibited earlier and more pronounced remodeling-associated activity, including increased matrix metalloproteinase-1 (MMP-1), MMP-2, MMP-9, and MMP/tissue inhibitors of metalloproteinases (TIMP), and enhanced ECM turnover, with higher production of collagen type III and fibrillin. These findings support the concept that GFs possess a more efficient remodeling phenotype, potentially contributing to the clinically observed rapid oral wound healing and reduced scarring. Similarly, in a recent study, we investigated how culture dimensionality modulates ECM remodeling behavior of GFs using a comparative 2D monolayer versus 3D fibrin hydrogel system (Ceylan et al., 2026). GFs embedded in 3D exhibited elongated, branched, web-like morphologies, in contrast to the spindle-shaped phenotype observed in 2D cultures, indicating dimensionality-dependent cytoskeletal organization. Histological analyses revealed progressive fibrin degradation accompanied by *de novo* collagen deposition within the 3D gels over a 21-day period. This remodeling was characterized by a time-dependent increase in MMP-2 activity, alongside downregulation of u-PA and t-PA at later stages, particularly in 3D cultures, suggesting a transition from early fibrinolytic degradation toward collagen-dominant matrix maturation. Collectively, these findings demonstrate that 3D fibrin hydrogels recapitulate key aspects of *in vivo* gingival wound healing and support their use as biologically relevant platforms to study periodontal soft tissue regeneration, fibroblast–ECM interactions, and mechanobiological regulation of wound repair.

Importantly, GFs also exhibit intrinsic osteogenic potential (Ceylan et al., 2024). Alfonso Garcia et al. (2022) demonstrated this potential using a scaffold-free 3D spheroid model by using a liquid overlay method using agarose. It was assessed whether human GFs can generate mineralization-associated features in the absence of exogenous osteoinductive supplements (Alfonso Garcia et al., 2022). Fibroblast identity in 2D and 3D were consistent with mesenchymal/fibroblast phenotypes (vimentin, COL1A2, PDGFRβ, integrin β1, αSMA), while spheroid culture induced stromal interaction molecule-1 (STIM1) expression which is linked to calcium entry/CRAC channel signaling, suggesting a mechanosensitive Ca^2+^-handling adaptation in 3D. Notably, spheroids developed surface-associated mineral-like microparticle deposits consistent with amorphous calcium phosphate, indicating that GFs in 3D can autonomously generate a microenvironment with mineralized niche formation. Collectively, these results support the use of GF for periodontal research and highlight fibroblast plasticity as a potentially functional feature for regenerative strategies.

## 6 Conclusion

Periodontium-derived fibroblasts provide a clinically relevant model to study periodontitis mechanisms, inflammatory bone remodeling, and therapeutic modulation, while supporting the development of more effective periodontal regenerative approaches. Their capacity to support both osteogenesis and osteoclastogenesis enables mechanistic investigation of bacterial triggers, inflammatory mediators, systemic comorbidities, and pharmacological interventions affecting periodontal bone balance. Future advances should focus on integrating these fibroblast-based assays into standardized 3D platforms that better mimic periodontal tissue architecture, ECM organization, and mechanical loading. Using multiple donors and functional assays may improve reproducibility and strengthen clinical relevance.
